# Inhibition of LXR**α**/SREBP-1c-Mediated Hepatic Steatosis by *Jiang-Zhi* Granule

**DOI:** 10.1155/2013/584634

**Published:** 2013-05-16

**Authors:** Miao Wang, Shanshan Sun, Tao Wu, Li Zhang, Haiyan Song, Weiwei Hao, Peiyong Zheng, Lianjun Xing, Guang Ji

**Affiliations:** ^1^Institute of Digestive Disease, Longhua Hospital, Shanghai University of Traditional Chinese Medicine, Shanghai 200032, China; ^2^Center of Chinese Medicine Therapy and Systems Biology, Shanghai University of Traditional Chinese Medicine, Shanghai 201203, China; ^3^E-Institute of Shanghai Municipal Education Commission, Shanghai University of Traditional Chinese Medicine, Shanghai 201203, China

## Abstract

Nonalcoholic fatty liver (NAFL) is increasingly recognized as one of the most common causes of chronic liver disease worldwide. Traditional Chinese medicine (TCM), as the alternative and complementary medicine, may provide some profound health benefit. “*Jiang-Zhi*” Granule (JZG) was composed based on TCM pathogenesis of NAFL: the retention of inner dampness, heat and blood stasis. This study investigated effects of JZG on liver X receptor-*α* (LXR*α*)/sterol regulatory element binding protein-1c (SREBP-1c) pathway in high-fat-diet-(HFD-)induced hepatic steatosis, as well as in free-fatty-acid-(FFA-)and T0901317-treated HepG2 cells. The results showed that JZG had an antisteatotic effect on HFD-fed rats. JZG decreased the activation of SREBP-1c through inhibiting LXR*α*-mediated SREBP-1c transcription, as well as through inhibiting the maturation of SREBP-1c independent of LXR*α*. These findings may provide molecular evidence for the use of JZG as a promising therapeutic option for NAFL and support us to continue JZG treatment in NAFL. For JZG treatment to be widely accepted, a randomized, double-blind, multicenter, placebo-controlled, phase III trial is ongoing.

## 1. Introduction 

Nonalcoholic fatty liver (NAFL) includes a spectrum of clinic pathological conditions with the characteristic change of excessive triglyceride accumulation in hepatocytes. NAFL is increasingly recognized as one of the most common causes of chronic liver disease worldwide [1] with the prevalence of about 15%–30% in the general population of various countries [2] and approximately 20% in china [3, 4]. Although generally patients with NAFL can live for decades without any clinically significant symptoms, they are at high risk of progressing to nonalcoholic steatohepatitis (NASH), cirrhosis, and ultimately hepatocellular carcinoma [5, 6]. Therefore, an effective pharmacological intervention is entailed to prevent or delay the onset and progress of NAFL. 

To date, no effective conventional western medicine is accepted as standard treatment to treat NAFL and its complications. Traditional Chinese medicine (TCM), as the alternative and complementary medicine, may provide some profound health benefit. In TCM algorithm, obesity-related diseases, including NAFL, are rooted in spleen Qi deficiency and inner phlegm retention; however, our previous study identified some limitations in treating NALF by method of spleen Qi-fortifying and phlegm resolving [7]. An appropriate and effective TCM treatment is based on a valid TCM theory. Epidemiological evidence [8] indicated that the crucial TCM pathogenesis of NAFL was the retention of inner dampness, heat and blood stasis. Thus a rationale NALF treatment should include the following elements: dampness-resolving, heat clearing, blood circulation activation, and stasis resolving.* Jiang-Zhi *Granule (JZG) was developed in accordance with the aforementioned TCM theory. It was composed of five herbs: Radix et Rhizoma Salviae Miltiorrhizae (24 g), Folium Nelumbinis (6 g), Rhizoma Polygoni Cuspidati (60 g), Herba Artemisiae Scopariae (6 g), and Herba seu Radix Gynostemmatis Pentaphylli (60 g). The quality was controlled under ultra-performance liquid chromatography (UPLC) as previously described [9]. JZG showed positive drug safety evaluation and obtained approval by State Food and Drug Administration (SFDA) for clinical trials (the Authorized Number is Z10960082). Our preliminary study demonstrated that JZG was beneficial for improving hepatic fat accumulation in HFD-fed rats [10]. We therefore conducted this study to explore whether JZG exerts a protective effect in NAFL, as well as its underlying mechanism. 

## 2. Materials and Methods

### 2.1. Animals and Interventions

Sprague-Dawley (200–250 g) rats were obtained from SLAC Laboratory Animals Co. (Shanghai, China). Rats were housed in a temperature of 22°C ± 2°C and humidity-controlled (50% ± 5%) room with a 12 hr light-dark cycle. Food (standard diet, STD, SLAC Laboratory Animals Co.) and drinking water were supplied ad libitum. Studies began after an acclimation period of one week. Animals were randomly divided into three groups: HFD+JZG group received 8-week high-fat diet (HFD consists of 10% lard oil, 2% cholesterol, and 88% STD) and then 4 weeks of HFD with JZG (*n* = 10), JZG was dissolved in saline and administered daily by oral gavage at a dose of 828 mg/kg/d, which was approximately 10 times of the standard dose in practice; HFD group received 8-week HFD and then 4 weeks of HFD with an equal volume of saline (*n* = 10); control group received 8-week STD and then 4 weeks of STD with an equal volume of saline (*n* = 10). We weighted the rats and recorded their food intake during the experimental period. 

At the end of the experimental period, blood samples were obtained from the abdominal aorta while rats were under anesthesia, livers were excised and weighed, and samples were either immediately snap-frozen in liquid nitrogen (for real-time PCR, western blot and hepatic TG measurement) or fixed in 4% PFA (for histological examination). 

All animal procedures were reviewed and approved by the Animal Experiment Ethics Committee of Shanghai University of Traditional Chinese Medicine. 

### 2.2. Plasma Biochemical Analysis

Plasma levels of triglyceride (TG), total cholesterol (TC), alanine aminotransferase (ALT), and aspartate transaminase (AST) were analyzed by an automatic blood chemistry analyzer (HITACHI 7170S, Japan).

### 2.3. Determination of Hepatic and Intracellular Lipid Content

Liver samples were fixed in 4% PFA, processed, and embedded into paraffin blocks, and then routine Hematoxylin and Eosin (H&E) stains were performed. Cells were fixed in 4% PFA for 30 min, washed in PBS, stained in Oil Red O for 20 min at room temperature, and then rinsed with PBS. Images were acquired on an Olympus BX-50 microscope.

Total liver lipid extracts were prepared using Folch's method [11]. Briefly, liver tissues (~200 mg) were homogenized in 2 mL of PBS and extracted twice with 2 mL of a chloroform/methanol (v : v = 2 : 1) solution and then centrifuged at 6000 rpm for 10 min to obtain the organic substratum (lower phase), which was dried and then resolubilized in 1 mL of chloroform. The mixed solution was used for measurement of triglyceride in duplicate, using the triglyceride (GPO-Trinder) kit as described by the manufacturer (Sigma, St. Louis, MO, USA).

### 2.4. Cell Culture

HepG2 cells were obtained from the Cell Bank of the Chinese Academy of Sciences (Shanghai, China). HepG2 cells were cultured in DMEM supplemented with 10% fetal bovine serum, 100 U/mL penicillin, 100 *μ*g/mL streptomycin, and 5.5 mmol/L D-glucose. Cells were incubated at 37°C in a 5%  CO_2_/95% air atmosphere on 100 mm diameter dishes, and analysis began after reaching 70% confluence.

For cell viability assay, cells were seeded in 96-well plates at a density of 1 × 10^4^ cells/mL in 100 ul culture medium. After 24 hours incubation, cell adherence was observed and the medium was refreshed with different concentrations of JZG (0, 5, 10, 50, 100, 500, and 1000 *μ*g/mL). Cell viability was determined by WST-1 assays (C0036, Beyotime, China) according to the manufacturers' instructions. 

 For experiments, cells were incubated on 12-well plates at a concentration of 1 × 10^5^ cells/mL or on 6-well plates at a concentration of 5 × 10^5^ cells/mL. Two HepG2-cell models were established: (1) HepG2 incubated in culture medium containing T0901317 (T090) (1 *μ*M) and treated with or without JZG (100 *μ*g/mL) for 24 hours [12]; (2) HepG2 cells incubated in culture medium containing palmitate (0.5 mM) and treated with or without JZG (100 *μ*g/mL) for 24 hours [13]. All experiments were performed independently in triplicate.

### 2.5. Preparation of Freeze-Dried Serum Containing Drugs

Drug-containing serum was obtained to study the pharmacological activity of herbs* in vitro*. Ten SD (200–250 g) rats were randomly assigned to accept JZG treatment or saline treatment (oral gavage). JZG was dissolved in saline and administered daily for one week by oral gavage at a dose of 828 mg/kg/d. Saline group were administered with an equal volume of saline for one week. One hour after the final treatment, all animals were anesthetized, and blood samples were obtained from the abdominal aorta under aseptic condition blood samples were then centrifuged at 3000 rpm for 15 min. All serums were filtered through a 0.45 *μ*m filter membrane and then concentrated and freeze-dried to achieve powder; all of these procedures were conducted by School of Pharmacy, East China University of Science and Technology (Shanghai, China). The powder was stored at −20°C until use.

### 2.6. Real-Time PCR

Total RNA was isolated from liver tissues and cells by the acid guanidinium thiocyanate/phenol/chloroform method, as described previously [14]. cDNA was prepared by 1 *μ*g of total RNA (ReverTra Ace qPCR RT Kit, Toyobo, Japan). Real-time PCR was performed using an ABI step one plus, real-time PCR System (Applied Biosystems, Foster City, CA, USA) with the SYBR Premix Ex Taq (SYBR Green Real-time PCR Master Mix,TOYOBO, Japan). The primers used were shown in Table 1.

### 2.7. Western Blot

Protein extraction (10 *μ*g) from liver tissues and cells was separated by SDS-PAGE. Immunoblotting was performed as described previously [14]. Protein expression was quantified using the Fujifilm Image Reader LAS-3000 (Fuji Medical Systems, Stamford, CT, USA) and NIH ImageJ software (http://rsbweb.nih.gov/ij/). Monoclonal anti-LXR*α*, anti-SREBP-1, anti-FAS, and anti-Actin antibodies were purchased from abcam.

### 2.8. Transient Transfection with siRNA

siRNA transfection was performed using Lipofectamine-RNAiMAX reagent (Invitrogen, Carlsbad, CA, USA) according to the manufacturer's instructions. Reverse transfection of HepG2 cells with siRNA (15 pmol/1.9-cm dish) targeting LXR*α* (Table 1) or with nonsilencing control siRNA (Invitrogen, Carlsbad, CA, USA) was performed. Cells were harvested after transfection to determine the mRNA and protein expression.

### 2.9. Statistical Analyses

Data were expressed as mean ± SD unless otherwise specified and evaluated using One-way Analysis of Variance (ANOVA), followed by Bonferroni post hoc test if a significant difference was detected by ANOVA. *P*  values < 0.05 were considered statistically significant. Statistical analyses were performed using SPSS 16.0 software (SPSS, Chicago, USA).

## 3. Results

### 3.1. Effects of JZG on HFD-Induced Body Weight and Liver Weight Gain

Rats were randomized by body weight. After 8 weeks of HFD feeding, body weight gain and liver/body weight ratio in the HFD group was higher than that in the control group (Table 2, *P* < 0.01). Four-week JZG treatment significantly reduced the body weight gain and liver/body weight ratio (Table 2, *P* < 0.05). Overall food intake did not differ among groups throughout this long-term experiment (data not shown). These results suggested that JZG could reduce HFD-induced body weight and liver weight gain in rats.

### 3.2. Effect of JZG on Plasma and Hepatic Lipid Levels

To determine whether JZG has an antisteatotic effect, we analyzed the plasma and hepatic lipid levels. As shown in Table 3, plasma levels of TC and TG in the HFD group were significantly increased compared to the control group; JZG treatment markedly relieved these increases (*P* < 0.01). In addition, compared to the control group, ALT and AST, which are sensitive indicators of liver damage, significantly elevated in the HFD group, and a decline was seen in the HFD+JZG group. These results indicated that HFD induced liver damage and JZG provided protective effect for the HFD-induced liver injury (Table 3).

**Figure 1:**
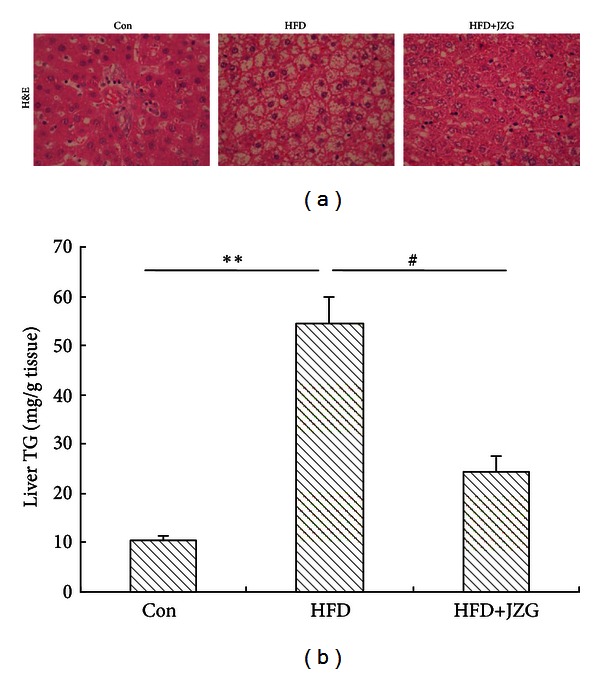
JZG reduced hepatic fat accumulation in the liver of HFD-fed rats. (a) Histological analysis of liver sections of HFD-fed rats treated with or without JZG and control group. Hematoxylin and eosin (H&E) staining. Original magnification ×400. (b) Triglyceride concentration in the liver. Data were presented as mean ± SEM. **P < 0.01 versus the control group, ^#^ *P* < 0.05 versus the HFD group.

Long-term HFD exposure induced not only increased plasma lipid, but also fat accumulation in the liver, leading to hepatic steatosis. Xu et al. showed that 8-week HFD induced more severe hepatic steatosis [15]. In this study, results from H&E staining showed that HFD feeding markedly increased lipid accumulation as shown by increases in both the number and size of liver fat droplets (Figure 1(a)). Consistent with hepatic histology, hepatic triglyceride content increased by 5.5-fold compared to that of the control group (Figure 1(b)), suggesting the development of fatty liver. In contrast, JZG treatment resulted in decreases in the lipid accumulation to some extent, and hepatic triglyceride content was similar to that in control group by concomitant JZG treatment.

The evaluation of plasma and hepatic lipid demonstrated that JZG could decrease lipid accumulation and alleviate HFD-induced fatty liver in rats.

### 3.3. Effect of JZG on Expression of Lipogenesis-Related Genes and Proteins

**Figure 2:**
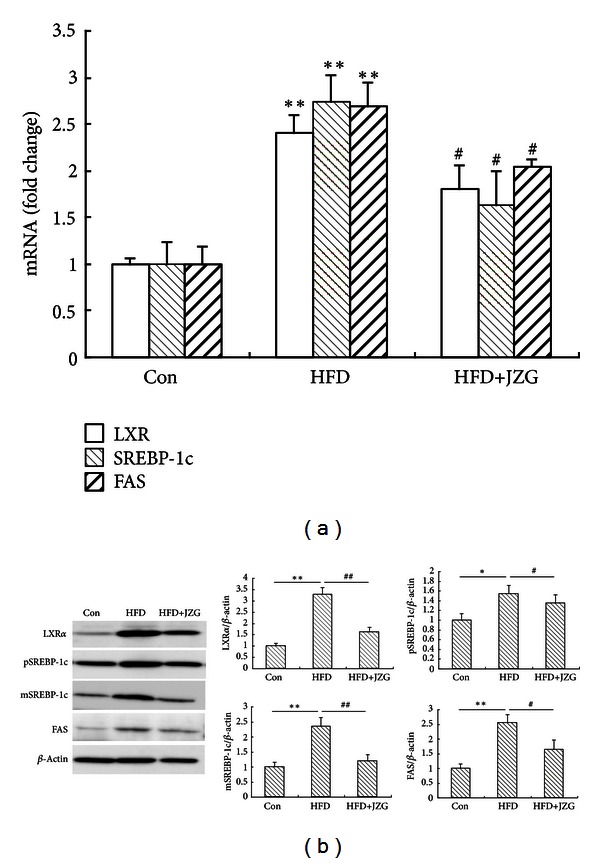
JZG reduced HFD-induced expression of lipogenesis-related genes and proteins. (a) The effect of JZG on HFD-induced LXR*α*, SREBP-1c, and FAS mRNA expression levels. (b) The effect of JZG on HFD-induced LXR*α*, pSREBP-1, mSREBP-1, and FAS protein expression levels. Data were presented as the mean ± SEM. *P < 0.05 and **P < 0.01 versus the control group, ^#^ *P* < 0.05 and ^##^ *P* < 0.01 versus the HFD group.

It has been reported that LXR*α*/SREBP-1c pathway plays a critical role in the regulation of hepatic lipid metabolism. In HFD-fed rodents, hepatic fat accumulation is closely related to the LXR*α*/SREBP-1c pathway [16, 17]. Therefore, we assessed the effect of JZG on LXR*α*/SREBP-1c pathway activation in the liver of HFD-fed rats. The mRNA expression of LXR*α*, SREBP-1c, and FAS changes in liver was shown in Figure 2(a). Compared to the control group, mRNA expression of these genes was significantly upregulated in the HFD group; HFD-induced upregulation of these genes was decreased by concomitant JZG treatment (Figure 2(a)).

In accordance with the changes in the mRNA expression, protein expression levels of hepatic LXR*α*, SREBP-1c precursor (pSREBP-1c), mature SREBP-1c (mSREBP-1c), and FAS were significantly increased in the HFD group, compared to the control group. As expected, these increases were significantly attenuated by JZG treatment (Figure 2(b)). 

Taken together, our results demonstrated that JZG suppressed hepatic steatosis through regulating the expression of genes and proteins related to lipogenesis.

### 3.4. Effects of JZG (Drug-Containing Serum) on FFA-Induced Lipid Accumulation in HepG2 Cells

 For cell viability assay, JZG at 500 and 1000 *μ*g/mL significantly decreased the cell viability, and concentration of 100 *μ*g/mL displayed no cytotoxic effects on the cells (Sup Figure 1) (see Supplementary material available online at http://dx.doi.org/10.1155/2013/584634). Therefore, 100 *μ*g/mL dose was selected for further study.

**Figure 3:**
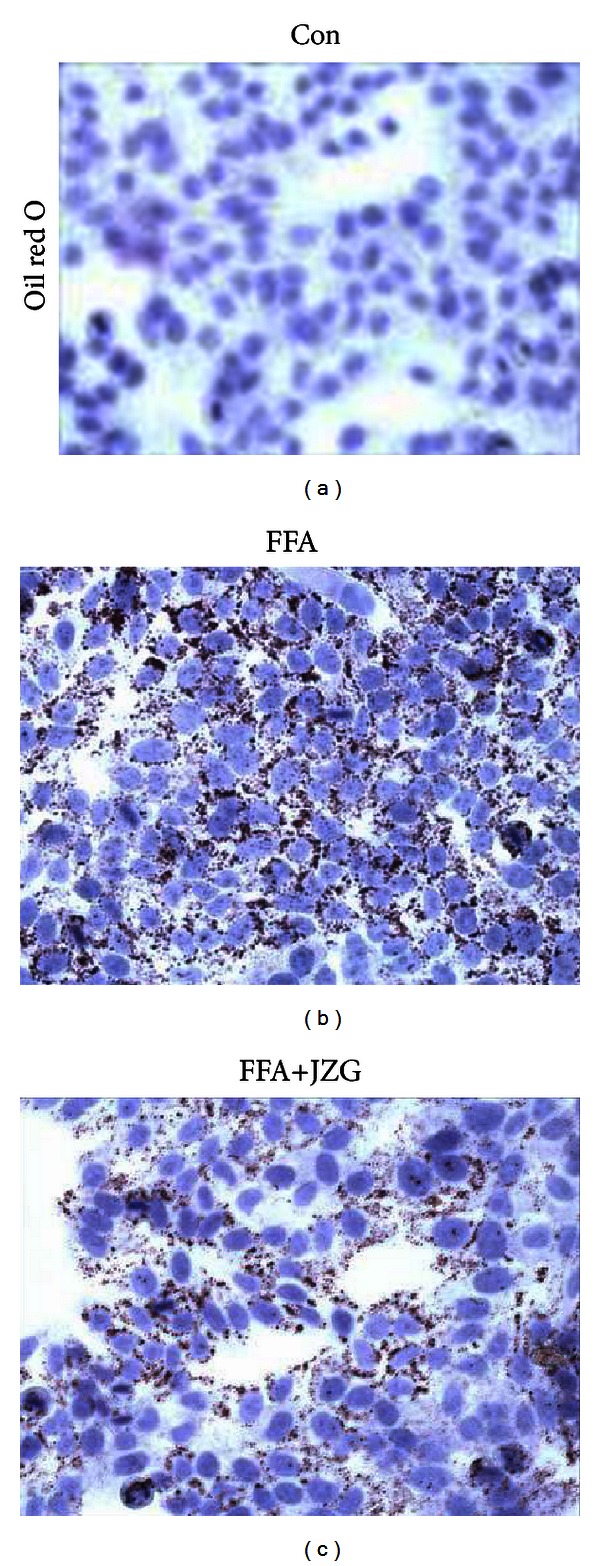
JZG decreased intracellular lipid content in HepG2 cells. Images showed lipid accumulation in cells stained by oil red O. Original magnification ×200.

As shown in Figure 3, consistent with the in vivo results, JZG reduced intracellular lipid accumulation. 

### 3.5. Inhibition of LXR*α* Activation by JZG (Drug-Containing Serum) in HepG2 Cells

**Figure 4:**
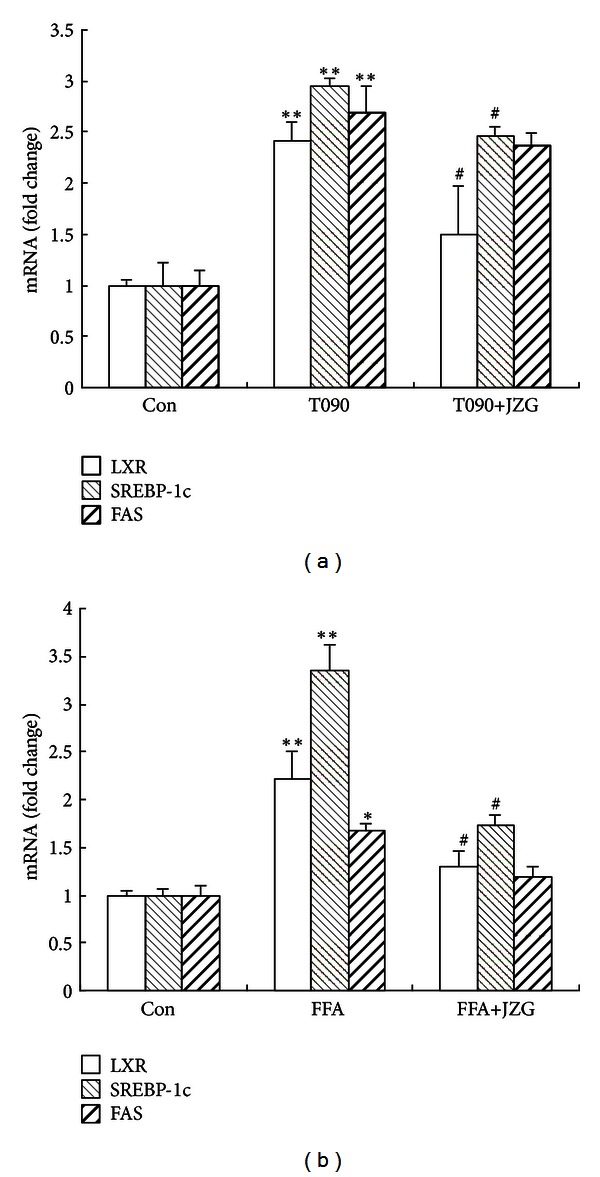
JZG reduced T090-mediated and FFA-induced expression of lipogenesis-related genes. (a) The effect of JZG on T090-mediated LXR*α*, SREBP-1c, and FAS mRNA expression levels. (b) The effect of JZG on FFA-induced LXR*α*, SREBP-1c, and FAS mRNA expression levels. The data represent the mean ± SEM of three separate measurements. *P < 0.05 and **P < 0.01 versus the control group, ^#^ *P* < 0.05 versus the FFA group.

SREBP-1c is a critical transcriptional factor which regulates hepatic lipogenic pathway, and LXR*α* is important in SREBP-1 expression and SREBP-1-mediated lipogenesis. We first investigated the effect of JZG on the T090-mediated and FFA-induced LXR*α*/SREBP-1c activation. The mRNA expression of LXR*α*, SREBP-1c, and FAS was significantly increased after treatment with T090 (1 *μ*M) or palmitate (0.5 mM) for 24 hours; this induction was inhibited by concomitant JZG treatment (Figure 4). 

Therefore, we speculated that JZG might inhibit the activation of LXR*α* and then decrease the expression of SREBP-1c and its target gene, FAS.

### 3.6. Inhibition of SREBP-1c Activation by JZG (Drug-Containing Serum) in siRNA Targeting LXR*α* HepG2 Cells

**Figure 5:**
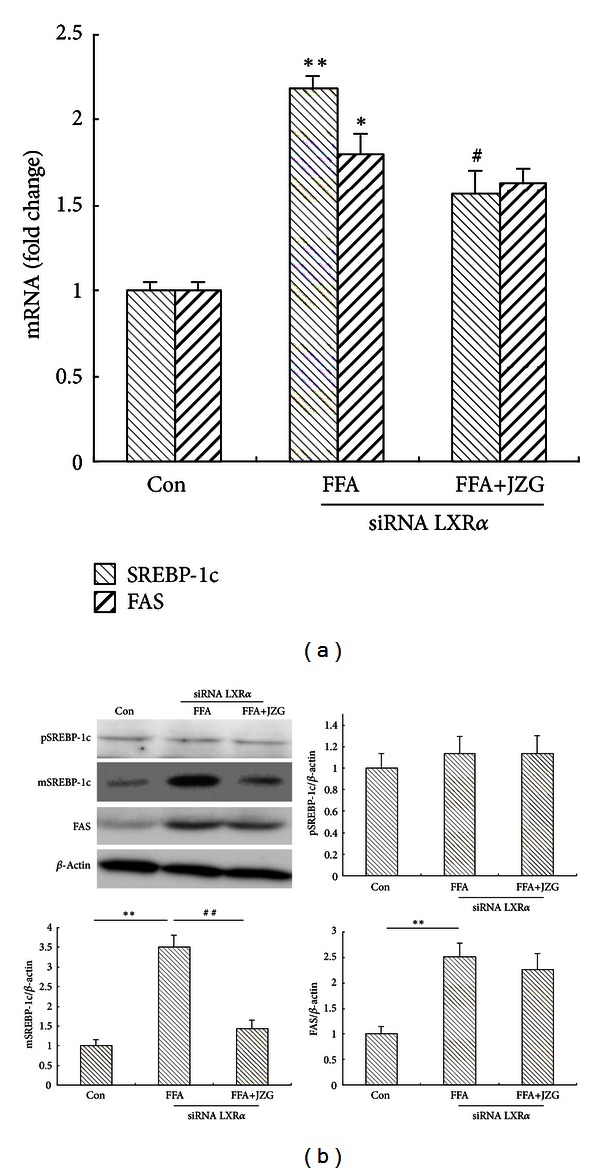
JZG reduced expression of SREBP-1c-related genes and proteins independent of LXR*α*. (a) The effect of JZG on FFA-induced SREBP-1c and FAS mRNA expression levels in LXR*α* knockdown HepG2 cells. (b) The effect of JZG on FFA-induced pSREBP-1c, mSREBP-1c, and FAS protein expression levels in LXR*α* knockdown HepG2 cells. The data represent the mean ± SEM of three separate measurements. *P < 0.05 and **P < 0.01 versus the control group, ^#^ *P* < 0.05 and ^##^ *P* < 0.01 versus the FFA group.

Some agents have little effect on the level of hepatic SREBP-1c precursor but significantly increase the level of hepatic mature SREBP-1c [18]. We have proved that LXR*α*-mediated elevation of SREBP-1c was alleviated by JZG treatment, but we had little knowledge about the effect of JZG on SREBP-1c activation independent of LXR*α*. Therefore, we introduced siRNA targeting LXR*α* in FFA-treated HepG2 cells to assess the activation of SREBP-1c. The siRNA targeting LXR*α* markedly reduced the levels of LXR*α* mRNA (sup Figure 2). As shown in Figure 5(a), in FFA-treated LXR*α* knockdown HepG2 cells, the mRNA levels of SREBP-1c and FAS were mildly increased compared to those in the control cells. However, these effects were blocked by concomitant JZG treatment. For protein examination, although JZG had little effect on the level of pSREBP-1c, the level of mSREBP-1c was significantly decreased (Figure 5(b)). Based on these results, we speculated that JGZ might not only decrease the expression of SREBP-1c through inhibiting LXR*α* activation, but also inhibit the maturation of SREBP-1c independent of LXR*α*.

## 4. Discussion

 To determine the effects of JZG on hepatic steatosis, two approaches were employed in this study: (1) an *in vivo* model involving HFD-fed rats and (2) an *in vitro* model by treatment with FFA, LXR*α* agonist (T090), and siRNA targeting LXR*α*. These results suggested that JZG exerted an anti-fatty liver effect through inhibition of LXR*α*/SREBP-1c pathway.

TCM has been used in China for thousands of years. It is widely practiced nowadays to manage different diseases and is regarded as an important part of the current health care system. Five herbs in JZG formula are generally used in China and listed in the Chinese Pharmacopoeia (2005 edition). They have good biological activities in lowering lipid level and improving liver function, as well as anti-inflammatory properties [19–21]. Integration of several herbs in a certain proportion to form a formula is the unique feature of TCM. The efficacy of a formula derives from the complex interactions of herbs, and this surpasses a single drug while treating certain disease. Therefore, we expected that JZG formula would achieve a maximized efficacy though potential synergistic effects of the five herbs. In our previous Randomized Controlled Trail (RCT) [22], 144 eligible patients were randomized to treatment group (JZG combinated with behavioral intervention) or control group (behavioral intervention alone). The primary outcome was an improvement in hepatic steatosis. The liver fat content was measured by ultrasound and graded into normal, mild, moderate and severe degrees [23]. The effective treatment was defined as one or more degrees decline in ultrasound grading after treatment. Our results demonstrated that a total effective rate was 76.56% in the treatment group versus 52.54% in the control group (*P* < 0.01). Nevertheless, further phytochemical studies elucidating its mechanisms are necessary for JZG treatment to be widely accepted into mainstream medical practice. 

 Dietary fat is one of the most important environmental factors associated with NAFL. It was previously shown that HFD is a good strategy for inducing a NAFL model [15]. The rat model in this study confirmed successful fat accumulation in the liver after HFD feeding for 8 weeks. The changes in body weight or liver weight, plasma levels of triglyceride and cholesterol, and hepatic fat accumulation were significantly lowered in the HFD+JZG group compared with the HFD group. Meanwhile, food consumption did not significantly differ between the two groups. These results suggested that energy intake did not contribute to the significant anti-fatty liver effects of JZG. 

 We speculated that the hypolipidemic effects of JZG came from the altered hepatic lipid metabolism. It was reported that dietary fat content, independent from caloric intake, is a crucial factor in the development of hepatic steatosis [24], and saturated fat intake can activate SREBP-1c and LXR*α* [25–27]. LXR*α* is a key regulator of fatty acid synthesis in NALF [18, 28] and a member of the nuclear receptor superfamily, which regulates the expression of key proteins involved in lipid metabolism [29]. LXR*α* agonist is primarily considered beneficial in the treatment of atherosclerosis [30], but the induction of fatty liver and hepatic dysfunction has limited their clinical development [31]. LXR*α* agonist also increases the expression of SREBP-1c, which leads to increased hepatic triglyceride synthesis [32]. Therefore, researchers consider the LXR*α*/SREBP-1c pathway an attractive target for the prevention and/or treatment of steatosis in hepatocytes [33], and several lines of evidence indicate that the suppression or disruption of LXR*α* leads to the reduction of hepatic triglyceride synthesis and accumulation [12, 34]. The present study found that the expression of LXR*α* and SREBP-1c was upregulated in HFD-fed rats, as well as FFA- and T090-treated HepG2 cells; JZG significantly alleviated the upregulation of LXR*α* and SREBP-1c in liver and cells. 

 SREBPs are a family of transcription factors that consist of SREBP-1a, SREBP-1c, and SREBP-2 [35]. SREBP-1c is the major isoform in liver and stimulates several lipogenic enzymes involved in liver fatty-acid synthesis [36], such as the gene that encodes FAS [37]. Increased SREBP-1c levels have been found in patients with histologically diagnosed NAFLD [38] and HFD-induced obese rats [39]. Transgenic mice overexpressing SREBP-1c produced massive fatty livers owing to increased accumulation of cholesteryl esters and triglycerides [40]. Several studies demonstrated that the mature form of hepatic SREBP-1c was significantly increased in fatty liver models [18, 41]. We therefore assessed whether JZG decreased hepatic steatosis through reducing SREBP-1c levels and/or activities independent of LXR*α*. We observed that JZG decreased the expression of SREBP-1c mRNA and mSREBP-1c protein in FFA-treated LXR*α* knockdown HepG2 cells. These results suggested that JZG could inhibit the maturation of SREBP-1c independent of LXR*α*. 

 The results from this study support us to continue JZG treatment in NAFL. JZG also has to withstand the rigorous scrutiny of research like other novel treatments. This required scientifically accepted research methodologies to avoid overstating study results. A randomized, double-blind, multicenter, placebo-controlled, phase III trial with 24 weeks followup is ongoing. The primary outcome is an alleviation in hepatic steatosis on the basis of computed tomography scans. Improvements in plasma biochemical parameters are secondary outcome measures. We expect that this confirmatory study will support JZG to be used in clinical treatment of NAFL. 

## 5. Conclusion

 We conclude that the effect of JZG on hepatic steatosis is likely to be multifactorial: JZG decreases the activation of SREBP-1c through inhibiting LXR*α*-mediated SREBP-1c transcription, as well as through inhibiting the maturation of SREBP-1c independent of LXR*α*. These findings may provide molecular evidence, at least partially, for the use of JZG as a promising therapeutic option for NAFL. 

## Supplementary Material

In order to provide further evidences for this study, the following experiments were performed: (1) Effect of JZG on HepG2 cells viability, and (2) siRNA targeting LXR*α* in HepG2 cells. The results were demonstrated as supplementary figures.

## Figures and Tables

**Table 1:** List of primers used in the present study.

Gene	Forward primer	Reverse primer
Rat LXR*α*	TCAAGGGAGCACGCTATGTC	GTTCCTCTTCTTGCCGCTTC
Rat SREBP-1	GGTTTTGAACGACATCGAAGA	CGGGAAGTCACTGTCTTGGT
Rat FAS	GGCACTGACTGTCTGTTTTCCA	GTAAAAATGACACAGTCCAGACACTTC
Human LXR*α*	GAGTTTGCCTTGCTCATTGC	ATCCGTGGGAACATCAGTCG
Human SREBP-1	CAGCCCCACTTCATCAAGG	ACTGTTGCCAAGATGGTTCCG
Human FAS	ACTGTTGCCAAGATGGTTCCG	GGCATCAAACCTAGACAGGTC
siRNA human LXR*α*	CGGAACAACTGGGCATGATCGAGAA	CGGCAACTGGGCATGATCGAAAGAA

**Table 2:** Physiologic and hepatic parameters in rats.

Group	Body weight (g)			Liver weight (g)	Liver/body ratio (%)
	Initial	Final	Gain		
Control (*n* = 10)	209.9 ± 6.5	323.8 ± 19.7	113.8 ± 13.6	8.6 ± 0.74	2.65 ± 0.10
HFD (*n* = 10)	211.5 ± 9.1	349.8 ± 25.2*	138.3 ± 17.7**	13.1 ± 1.45**	3.75 ± 0.18**
HFD+JZG (*n* = 10)	212.1 ± 7.7	333.0 ± 23.4	120.9 ± 17.3^#^	11.7 ± 1.86^#^	3.48 ± 0.33^#^

HFD: high-fat diet, JZG: “*Jiang-Zhi*” Granule.

*P < 0.05 and  **P < 0.01 versus the control group, ^#^ *P* < 0.05 versus the HFD group.

**Table 3:** Plasma biochemical parameters in rats.

	Control (*n* = 10)	HFD (*n* = 10)	HFD + JZG (*n* = 10)
TC (mmol/L)	1.44 ± 0.14	1.81 ± 0.81**	1.55 ± 0.55^##^
TG (mmol/L)	0.73 ± 0.09	1.43 ± 0.11**	1.01 ± 0.15^##^
ALT (U/L)	19.66 ± 1.50	30.01 ± 2.73**	25.50 ± 4.55^##^
AST (U/L)	6.62 ± 0.42	20.75 ± 2.59**	12.09 ± 1.69^##^

**P < 0.01 versus the control group, ^##^ *P* < 0.01 versus the HFD group.
