# Supplementary material for: Inhibition of LXRα/SREBP-1c-Mediated Hepatic Steatosis by Jiang-Zhi Granule
Source: Evid Based Complement Alternat Med. 2013 May 16;2013:584634. doi: 10.1155/2013/584634 (PMC3670567; doi:10.1155/2013/584634)
Supplement: Supplementary file 1 — In order to provide further evidences for this study, the following experiments were performed: (1) Effect of JZG on HepG2 cells viability, and (2) siRNA targeting LXRα in HepG2 cells. The results were demonstrated as supplementary figures. [file 584634.f1.doc]

**SupFig. 1 Effect of JZG on HepG2 cells viability.**

HepG2 cells were treated with HepG2 at different concentration (0, 5, 10, 50, 100 500 and 1000 mg/L). WST-1 experiments were conducted after 6, 12, 24 and 48 hours.

**A B**


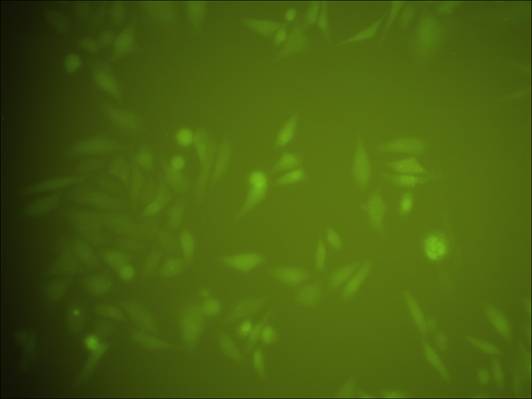


**supFig. 2 RNAi in HepG2 cells**

(A) siRNA uptake assessment by BLOCK-iT^MT^ Fluorescent Oligo in HepG2 cells. Original magnification x 200.

(B) siRNA targeting LXRα in T090-induced HepG2 cells.
